# Interventions to Prevent Relapse or Recurrence of Preconception Anxiety and/or Depression in Perinatal Women: A Systematic Review

**DOI:** 10.1007/s10995-025-04054-1

**Published:** 2025-01-23

**Authors:** Celia Rae, Rebecca McRae, Elizabeth Holliday, Catherine Chojenta

**Affiliations:** School of Medicine and Public Health, University of Newcastle, Newcastle, NSW Australia

**Keywords:** Relapse, Recurrence, Anxiety, Depression, Antenatal, Postnatal, Perinatal

## Abstract

**Objectives:**

Women with preconception anxiety and/or depression experience high rates of relapse or recurrence of the disorders in the perinatal period. This review aimed to identify perinatal interventions that were designed to prevent relapse or recurrence in women with a history of anxiety and/or depression.

**Methods:**

The review was conducted based on the PRISMA guidelines. Six medical databases were searched with specific search strategies for each. The reference lists of literature reviews retrieved in this search were also screened, as well as the reference lists of reviews identified within these reviews. Additionally, the publications of the first authors of included studies were reviewed for relevant articles.

**Results:**

There were 10 articles eligible for inclusion. These articles described pharmacological or dietary supplement interventions, as well as psychological and/or behavioural interventions. All identified studies focused on the prevention of recurrent depression, comprising four antenatal interventions and six postnatal interventions. No studies reporting interventions for the prevention of recurrent anxiety were identified.

**Conclusions for Practice:**

Several published studies provided evidence supporting the use of prophylactic antidepressants and progesterone to prevent relapse or recurrence of depression, although studies were limited by small sample sizes and the potential for study bias. More recent and higher quality evidence exists for the role of mindfulness and cognitive behavioural therapy in the prevention of depressive relapse. Further exploration of relapse prevention strategies for women with preconception anxiety and/or depression is required, particularly for recurrent anxiety.

**Supplementary Information:**

The online version contains supplementary material available at 10.1007/s10995-025-04054-1.

## Significance

What Is Already Known on This Subject?

What This Study Adds?

Preconception mental health difficulties is one of the most consistent predictors of perinatal anxiety and/or depression. Rates of perinatal anxiety and/or depression are also much higher for women with a prior history of the disorders. As the significant, negative impacts of untreated perinatal anxiety and/or depression on the health and development of offspring are well established, the prevention of these disorders in women who are at heightened risk is a priority.

This review provides information for clinicians and allied health providers to consider when caring for perinatal women with preconception anxiety and/or depression, with the aim of preventing perinatal relapse/ recurrence.

**Supplementary Information:**

The online version contains supplementary material available at 10.1007/s10995-025-04054-1.

## Introduction

It is estimated that nearly 12% of women experience perinatal depression worldwide (Woody et al., 2017). This figure likely represents an underestimate of the actual burden of disease, given that many affected women may either not recognise their symptoms or not seek treatment (Pereira et al., 2014) and there is a paucity of prevalence and incidence data from low- and middle-income countries (Woody et al., 2017). The prevalence of perinatal anxiety is even higher, with nearly 21% of women estimated to experience at least one type of anxiety disorder during the perinatal period (Fawcett et al., 2019). A considerable number of women also experience comorbid anxiety and depression, with prevalence estimates between 4.6 to 47.6% reported during pregnancy (Bante et al., 2021; González-Mesa et al., 2020; Hou et al., 2023; Thiagayson et al., 2013) and 7.1 to 13% postnatally (Falah-Hassani et al., 2016; Hou et al., 2023).

Perinatal anxiety and depression can adversely impact foetuses and infants, the consequences of which can extend into childhood and adolescence. Evidence suggests antenatal anxiety alters infant brain structure and function (Adamson et al., 2018) and exposed newborns have a greater risk of negative emotional reactivity and self-regulatory difficulties (Korja et al., 2017). Postnatal anxiety has been associated with poorer bonding and reduced mother-infant interaction (Field, 2018).

Women experiencing antenatal depression may practise poor self-care, which can contribute to poor birth outcomes, including premature birth and low birth weight (Dadi et al., 2020; Ghimire et al., 2021; Uguz et al., 2019), and postnatal depression has been linked to increased risk of attention deficit/ hyperactivity disorder (ADHD) in children (Christaki et al., 2022), and mental health difficulties in childhood (Szekely et al., 2021) and adolescence (Verbeek et al., 2012). A recent meta-analysis of 191 studies (N = 195,751 mother–child dyads) reported negative associations of perinatal anxiety and depression with social-emotional, cognitive, language, motor and adaptive behavioural development of children (Rogers et al., 2020).

One of the strongest predictors of perinatal anxiety and/or depression is mental health difficulties before pregnancy (Austin et al., 2017; Field, 2018; Furtado et al., 2018; Hutchens & Kearney, 2020; Xueyan Liu et al., 2022a, 2022b; Martini et al., 2015; Míguez & Vázquez, 2021; Patton et al., 2015; Yin et al., 2021; Zhao & Zhang, 2020). Rates of perinatal anxiety and depression are higher in women with a history of these disorders with relapse rates reported as 20.8% for women who experienced depression before pregnancy and 56% for women who experienced anxiety (Martini et al., 2015). For women who experienced preconception comorbid anxiety and depression, the rate of perinatal relapse has been reported as 29.2% for depression and 63.1% for anxiety (Martini et al., 2015).

While women with preconception anxiety and/or depression are at heightened risk of perinatal mental health difficulties, the literature reports relatively few interventions targeting this group. A recent systematic review of psychological and psychosocial interventions, evaluated to determine their effectiveness in preventing common perinatal psychiatric disorders, identified nine universal interventions and 12 interventions for at-risk or targeted groups, such as adolescent or primiparous women (Waqas et al., 2022). Only one study targeted women with a history of anxiety and/or depression (Dimidjian et al., 2015).

The purpose of this review was to identify and synthesise the results of studies evaluating any intervention designed for women with a history of anxiety and/or depression and minimal to no symptoms immediately prior to pregnancy, to prevent relapse or recurrence of their past condition during the perinatal period. This review provides new information to clinicians, practitioners and researchers to consider when designing interventions to target women whose history of anxiety and/or depression places them at heightened risk of developing perinatal anxiety and/or depression.

## Methods

The systematic review was conducted according to the 2020 PRISMA guidelines (Page et al., 2021). Six databases were searched in the review: APA PsychINFO, Embase, Maternity and Infant Care Database, Medline, CINAHL and Scopus with specific search strategies formulated for each database. The search extended from database inception to the date the review was conducted (13th February 2023).

Included in the review were studies of women with a history of anxiety and/or depression before the index pregnancy, including postnatal anxiety and/or depression after previous children. Depression and anxiety were defined according to the DSM-V (APA, 2013) and included such conditions as generalised anxiety disorder and major depressive disorder. The presence of anxiety or depression may have been established via self-report, clinical assessment, or any other validated measures.

To focus on an outcome of relapse, women must have reported minimal to no symptoms of anxiety and/or depression at the start of the intervention, which was established by the authors describing participants as being well. For studies that included standardised measures, such as the Edinburgh Postnatal Depression Scale (EPDS), minimal to no symptoms of depression were defined as a score less than 9, where ‘scores in this range may indicate the presence of some symptoms of distress that may be short-lived and are less likely to interfere with day-to-day ability to function at home or at work’ (Cox et al., 1987). For the Patient Health Questionnaire (PHQ-9), minimal to no depression was defined as a score of 0–4 (Kroenke et al., 2001).

Eligible interventions were any program, service or resource relevant to the review question that had undergone some form of evaluation, either formal or informal (e.g., participant feedback). There were no restrictions on content, start time or delivery mechanisms. Universal programs intended to prevent perinatal anxiety and depression could be included, provided they reported results separately for women with a history of anxiety and/or depression.

Outcomes of interest were staying well during the perinatal period versus relapse or recurrence of preconception anxiety and/or depression, as measured using validated tools such as the EPDS or PHQ-9, or self-report. No restrictions were placed on study design, with the inclusion of randomised studies, quasi-experimental and observational studies. Protocol papers were excluded, together with conference abstracts, posters, and presentations, the brevity of which can limit assessments of study quality. Only studies published in English were included in the review.

Titles and abstracts were screened by two reviewers (CR and RM) and any conflicts were resolved by discussion. The titles and abstracts of articles included in the reference lists of literature reviews were screened, as well as the titles from the reference lists of the literature reviews within literature reviews. For studies included in the review, other publications of the first author were also screened.

Full texts of articles were retrieved and analysed for eligibility. Of those deemed relevant, information on study design, participant characteristics, intervention, outcome measures and findings was extracted and presented in tabular form. Each of the articles underwent a quality assessment using either the JBI Critical Appraisal Tool for RCTs (Barker et al., 2023), case series (Munn et al., 2020) or quasi-experimental studies (Tufanaru et al., 2017). Bias was also assessed with the Cochrane Risk-of-Bias Tool for Randomized Trials (RoB-2) (Sterne et al., 2019) or the Risk Of Bias In Non-Randomized Studies of Interventions (ROBINS-1) (Sterne et al., 2016). The findings were synthesised to create a narrative summary.

## Results

In total, 4,032 records were retrieved from medical databases and an additional 3,830 articles were retrieved from relevant literature reviews amongst these records. Once duplicates were removed, the titles and abstracts of 5,283 articles were screened for relevance to the research question. The full text of 444 articles was obtained; the majority of these were subsequently excluded.

Reasons for exclusion included not considering women with preconception anxiety and/or depression as a distinct sub-group for analysis, for example, studies that did not measure or describe the psychiatric history of participants (e.g. Kavanagh et al., 2021; Tandon et al., 2018) and studies that did differentiate sub-groups, but only to measure and manage risk of confounding (Zlotnick et al., 2016). Other excluded studies combined women with preconception anxiety and/or depression with women with a family history of the disorders (e.g. Lewis et al., 2014) or a personal history of unspecified mental illness (Tachibana et al., 2019). Some studies were excluded because they analysed intervention effects for women with preconception anxiety and/or depression, combined with women with symptoms of distress that required monitoring (EPDS score 10–12, PHQ-9 score ≥ 4) (e.g. Felder et al., 2017; Le et al., 2021).

Literature reviews, protocol papers, conference abstracts, presentations and posters were also excluded, while one article (Dalton, 1989) was unable to be retrieved after extensive searching online, in medical databases and contacting corresponding authors.

**Fig. 1:**
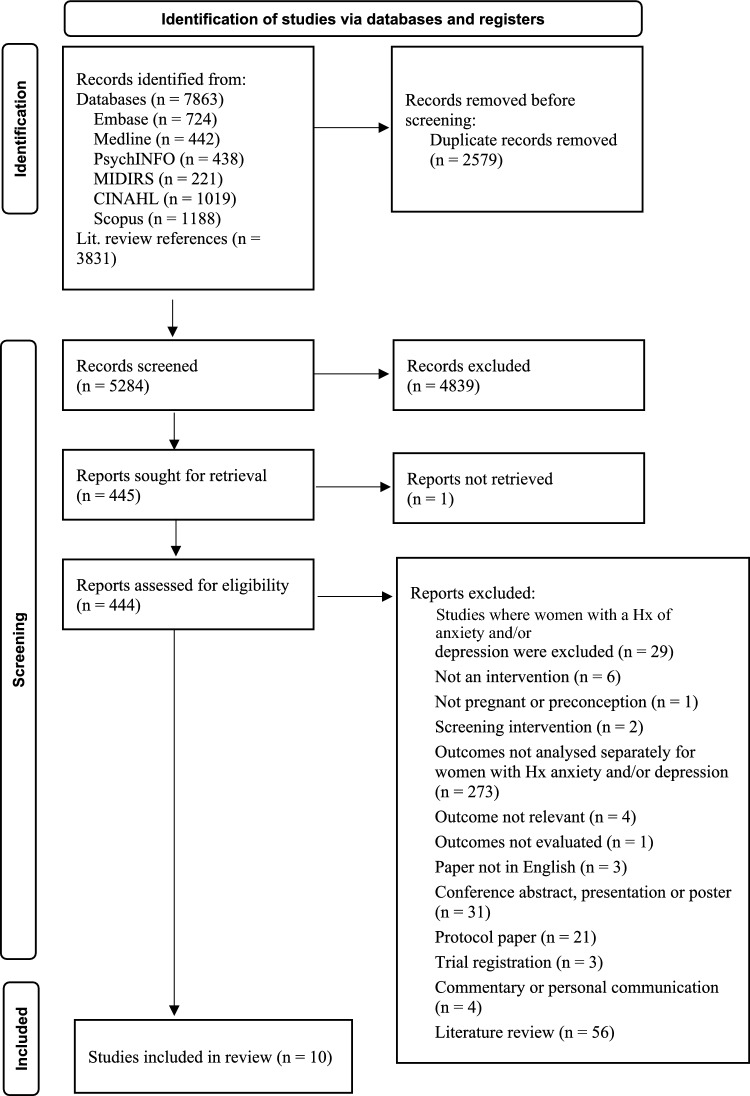
PRISMA Flow Diagram based on Page et al. (2021)

After the exclusion of 434 full texts, 10 papers were eligible for inclusion in the review. Information about the characteristics, effectiveness, quality and risk of bias was then extracted and summarised. The search process and reasons for exclusion are illustrated using the PRISMA flow diagram in Fig. 1.

### Participants

Amongst the included papers, sample sizes ranged from 7 to 450 with only one study including more than 100 participants. Participants were women aged between 20–40 years, most of whom were white and either married or in a long-term relationship. In antenatal studies, participants were between 5–32 weeks gestation at the beginning of the intervention and from birth to six weeks postpartum for postnatal interventions. Three of the studies reported the number of major depressive episodes participants had experienced before pregnancy (Dimidjian & Goodman, 2014; Dimidjian et al., 2015; Marangell et al., 2004) with one episode being the most common. Two studies included information about anxiety, stating that around one-third of participants reported experiencing the disorder (Dimidjian et al., 2015, 2016).

### Interventions

Of the 10 studies identified, seven were conducted in the United States with the others conducted in England (n = 1), Brazil (n = 1) and the Netherlands (n = 1). There were six randomised controlled studies (RCTs), three case studies and one open clinical trial. All studies focused on the prevention of recurrent depression during the perinatal period (as opposed to perinatal anxiety) with four antenatal interventions and six postnatal interventions. Six of the studies focused on preventing recurrent postnatal depression. The interventions varied in length from 2 to 10 months, with the last follow-up assessment between 3 and 6 months postpartum for most studies; the latest was at 16 months postpartum (Molenaar et al., 2020).

Studies comprised two types of interventions: pharmacological or dietary supplements (n = 6), centred on the prophylactic administration of antidepressants, progesterone or fish oil; and psychological and/or behavioural interventions (n = 4), including mindfulness, cognitive therapy and exercise. Most studies reported a primary outcome of relapse or recurrence of depression, measured using several tools, most commonly the EPDS and the Hamilton Depression Rating Scale (HAM-D). Other primary outcomes included prevalence, severity, and changes in depressive symptomatology, and time to recurrence. Secondary outcomes included satisfaction, acceptability and engagement with the intervention.

### Pharmacological and Dietary Supplementation

Prophylactic sertraline, a selective serotonin reuptake inhibitor (SSRI) antidepressant, was found to be efficacious with one small RCT (n = 7) reporting a recurrence rate of 7% in the treatment group compared to 50% in the placebo (Wisner et al., 2004). Administration of prophylactic antidepressants more broadly, including clomipramine, fluoxetine, imipramine and nortriptyline, produced similar results, with 6.7% of women experiencing recurrence in the treatment group compared to 68% in controls (Wisner & Wheeler, 1994). A subsequent study of prophylactic nortriptyline, however, found no effect on rates of recurrence (Wisner et al., 2001).

Prophylactic progesterone has gathered some support as a preventative postnatal depression strategy, with one study by Dalton (1985) reporting a recurrence rate of 10% compared to 68% in a previous study by the same author (1980).

Minimal to no effects have been demonstrated with omega-3 supplementation. One study by Vaz et al. (2017) reported a significant reduction in EPDS score from pregnancy to postpartum compared to a control group, but found no difference in rate of recurrence or mean of EPDS score. Another study evaluating omega-3 supplementation reported no effect, and was suspended after four of its seven participants relapsed within 12 weeks (Marangell et al., 2004).

### Psychological and/or Behavioural Interventions

One case series and one RCT assessed mindfulness-based cognitive therapy adapted for the perinatal period (MBCT-PD) and reported effectiveness for preventing recurrent depression in women with a history of major depressive disorder (Dimidjian et al., 2015, 2016). MBCT-PD in the case series comprised eight two-hour sessions focusing on mindfulness and cognitive behavioural skills, with the addition of psychoeducation, yoga and self-care in the RCT. Significantly lower rates of depressive relapse or recurrence were reported for participants in both studies (18% in case series, 18% MBCT-PD versus 50.2% usual care in RCT) together with significantly lower depressive symptom severity. Significant improvements in depressive symptom levels were also reported in the case series. Retention was high and participants reported interest, engagement and satisfaction with the program. Most participants in the case series reported that the program changed how they responded to intense emotions and helped them recognise and respond to early warning signs of relapse or recurrence.

A non-inferiority RCT by Molenaar et al. (2020) investigated the risk of depressive relapse in pregnant women with preconception major depression. The intervention group discontinued antidepressant medication in favour of preventative cognitive therapy (PCT), which uses cognitive challenging techniques focussed on dysfunctional beliefs and schema. The control group continued with pharmacological therapies. At the last follow-up visit of the study, no significant difference in risk of relapse was found between the treatment and control groups, indicating efficacy of the intervention for preventing depression, whilst offering pregnant women a non-pharmacological treatment option to minimise risk to the developing foetus.

In another study, exercise was shown to reduce perceived stress in participants, and a wellness and support intervention was found to decrease symptoms of depression (Lewis et al., 2021). However, no differences in rates of recurrence were found and none of the changes were sustained at 9 months follow-up.

More details of each study are available in Tables A.1 and A.2 in the supplementary material.

### Quality Assessment and Risk of Bias

Critical appraisal and risk of bias assessments were undertaken for each study and are presented in Tables A.3 to A.7 in the supplementary material. Most of the studies included in this review were randomised controlled trials (n = 6) that used established strategies to increase validity and minimise bias, including: blinding of participants (e.g. Vaz et al., 2017); blinding of personnel (e.g. Lewis et al., 2021); blinding of participants and personnel (e.g. Wisner et al., 2001, 2004); stratified randomisation of participants to reduce confounding bias (e.g. Molenaar et al., 2020); intention-to-treat analysis (e.g. Lewis et al., 2021) and assessment of interrater reliability in rating adherence to the treatment protocol (e.g. Dimidjian et al., 2016).

The other four studies were more prone to bias due to their study design. Three were case series, meaning causation could not be proven and the potential for confounding bias was increased (Dalton, 1985; Dimidjian et al., 2015; Marangell et al., 2004). Moderate risk of bias was introduced in the case series by Dimidjian et al. (2015) by not excluding women taking psychotropic medication and receiving psychotherapy at baseline, thereby meaning participants could potentially receive differing interventions during the study period.

The possibility of selection bias was introduced in the open clinical trial by Wisner and Wheeler (1994), by allowing participants to choose which arm of the study they were allocated to (antidepressant with monitoring versus monitoring alone). Women who had responded well to antidepressants in the past may have selectively chosen the intervention group, while women who did not may have preferred the control group, thus biasing the estimated intervention effect. Participants receiving antidepressants also received different antidepressants depending on which agents they had responded to in the past, thereby introducing further potential bias.

While the case series by Dalton (1985) could not be properly assessed due to the brevity of the article, there are some reported characteristics of the study that decrease validity and increase the risk of bias. Firstly, previous episodes of postnatal depression were not measured in a standard and reliable way, with episodes only requiring to be ‘severe enough to require medical treatment.’ No outcome measures were described, and recurrence was assessed face-to-face with half of the participants and via questionnaire with participants and/or their general practitioners for the other half of the group. Confirmation bias was possible given the author had used progesterone prophylaxis with pregnant women for many years beforehand. Furthermore, the conclusions reached by the author were also potentially unreliable, being based on comparison of recurrence rates from two cohorts of women from different studies.

For all the studies, generalisability is moderately low given the participants were predominantly white, married and college educated. Several studies also included very small sample sizes (n = 7, Martini et al., 2015; n = 22, Wisner et al., 2004; n = 23, Wisner & Wheeler, 1994), and thus had limited statistical power.

## Discussion

To the best of our knowledge, this study is the first review of perinatal interventions delivered with the aim of preventing relapse or recurrence in women with preconception anxiety and/or depression. Relatively few studies have evaluated interventions for this population, despite high rates of recurrence and established, long-term impacts of maternal anxiety and depression on children. This is particularly the case for women with a history of anxiety, for which no interventions to prevent relapse were identified. Perhaps because comorbid anxiety and depression is so prevalent (Hou et al., 2023), it is assumed that interventions to prevent depression are sufficient for women with preconception anxiety as well. However such interventions may inadvertently exclude women with a history of anxiety only, an estimated 56% of whom develop a recurrence of their condition perinatally (Martini et al., 2015).

In contrast, there are many universal interventions focusing on the prevention (rather than relapse) of perinatal anxiety and/or depression for all pregnant or postpartum women (Waqas et al., 2022). Inevitably, women with preconception anxiety and/or depression are participating in these interventions given an estimated 5% of women reported experiencing anxiety and 4.5% reported experiencing depression worldwide in 2019 (Institute of Health Metrics and Evaluation, 2023). As such, they will already be receiving mental health promotion and early intervention strategies from universal perinatal self-care interventions.

Yet it is likely the information needs and self-care strategies of women who have experienced preconception anxiety and/or depression are different. Instead of learning about the signs and symptoms of anxiety and depression, women who have already experienced the disorders might benefit more from focused interventions on modifying negative thought patterns and maladaptive coping strategies. The need for information about psychotropic medications and the risks and benefits of continuing them during pregnancy and breastfeeding is also different from other women who do not take medication. Determining early warning signs specific to each woman and creating action plans in consultation with health care providers if symptoms of mental health difficulties develop could also be a focus. While these examples are used to further the argument that women with preconception anxiety and/or depression have different health information needs, no such studies examining their needs or preferences for support were identified by either of two recent systematic reviews on the topic (Ghiasi, 2021; Lu et al., 2022), and further exploration through surveying and/or interviewing women with preconception anxiety and/or depression might be beneficial.

While acknowledging this review may have omitted relevant studies where the intervention was not evaluated or the findings published, among studies captured by the review, there emerged obvious gaps in the literature. In addition to the absence of anxiety relapse prevention studies, all pharmacological studies included in the review aimed to prevent a relapse of postnatal depression versus depression more broadly. While there are undoubtedly similarities in the mechanisms of developing both depression types, these two groups of women may respond differently to antidepressants, which further research could assess.

Studies evaluating prophylactic antidepressants and progesterone were all conducted before 2004. This presumably reflects the recent preference of women to refrain from medication use during pregnancy, and minimise potential impacts on their child (Knight et al., 2022). In a French nationwide cohort of 766,508 pregnancies, 68% of women who were taking antidepressant medication at the beginning of their pregnancy stopped treatment within the first trimester (Bénard‐Laribière et al., 2018). However, medication discontinuation has been associated with an increased risk of psychiatric emergency (Xiaoqin Liu et al., 2022a, 2022b) and relapse for women with a history of severe or recurrent depression (Bayrampour et al., 2020). Given limited evidence showing negative effects on birth weight, neurodevelopmental or neurobehavioural outcomes (Prady et al., 2018), prophylactic antidepressant use in pregnancy may be safe and effective for preventing recurrent anxiety and/or depression.

Two behavioural and psychological interventions identified by this review significantly and positively impacted symptoms of depression, as well as relapse or recurrence rates in women with a history of depression (Dimidjian et al., 2015, 2016). While these interventions may provide sufficient evidence to guide the design of future interventions for women with preconception depression, they do not consider preconception anxiety and they are time and resource-intensive to deliver, requiring a clinical psychologist and behavioural provider to attend a five-day training workshop in Mindfulness Based Cognitive Therapy and to deliver eight two-hour face-to-face sessions to participants. Women were also required to invest time by attending the intervention, which was not possible for all women, for example, those who worked full-time or cared for young children. The high resource intensity of these interventions means that they could not be delivered on a large scale without considerable financial investment, and it might be helpful to investigate other types of less resource-intensive interventions.

This review did not identify any interventions with long-term follow-up and assessments of outcomes. The last follow-up assessment for most of the studies was performed at 3 or 6 months postpartum, with the longest follow-up being at 16 months postpartum reported by Molenaar et al. (2020). The longer term impacts of these interventions, while difficult to capture for pragmatic reasons, thus remain understudied. For example, that the exercise and wellness intervention by Lewis et al. (2021) showed significant differences in symptoms of depression and perceived stress between groups at 6 months, but these differences were not significant at 9 months. Longer follow-up periods – for example, up to 5 years – would provide more comprehensive evidence regarding long term intervention effects, and also the effects of interventions on subsequent pregnancies.

A strength of this study is the comprehensive nature of the review. The search strategy included the screening of 5,283 titles and abstracts and 444 full texts. The reference lists of 56 literature reviews were searched, as well as the reference lists of the literature reviews included in these reviews. In addition, the publications of the lead authors of each study were screened. A limitation is that only one reviewer screened the references of the systematic reviews and the full text of articles.

## Conclusion

Women who have experienced preconception anxiety and/or depression have increased risk of relapse or recurrence during the perinatal period. Despite this risk, relatively few selective prevention studies have been reported. There is evidence to support the use of prophylactic antidepressants and progesterone, though these studies are limited by small sample sizes and the potential for bias. There is more recent and comparatively higher quality evidence to support the effectiveness of mindfulness and cognitive behavioural therapy, though these interventions also had small sample sizes and were time and resource-intensive.

Further exploration of prevention strategies is required, particularly for recurrent anxiety, together with research on how to deliver effective interventions with limited resources. Research to understand the health information needs and support preferences of women with preconception anxiety and/or depression might also be investigated.

## Supplementary Information

Below is the link to the electronic supplementary material.

Supplementary file1 (DOCX 61 KB)

## Data Availability

Not applicable.
