# Supplementary material for: Interventions to Prevent Relapse or Recurrence of Preconception Anxiety and/or Depression in Perinatal Women: A Systematic Review
Source: Matern Child Health J. 2025 Jan 23;29(3):294–303. doi: 10.1007/s10995-025-04054-1 (PMC11926042; doi:10.1007/s10995-025-04054-1)
Supplement: Supplementary file 1 — Supplementary file1 (DOCX 61 KB) [file 10995_2025_4054_MOESM1_ESM.docx]

# Supplementary material

Interventions to prevent relapse or recurrence of preconception anxiety and/or depression in perinatal women: A systematic review; Maternal Child Health Journal; Celia Rae, Rebecca McRae, Elizabeth Holliday, Catherine Chojenta; University of Newcastle; celia.rae@newcastle.edu.au

## Table 1: Pharmacological and dietary supplement studies

| **Study; country** | **Study design; period; prevention focus** | **Hypothesis/aim** | **Sample (n); participant characteristics** | **Intervention description** | **Outcomes and measures** | **Findings** |
| --- | --- | --- | --- | --- | --- | --- |
| Dalton, 1985; England | Case series; postnatal; PND | To evaluate the effectiveness of prophylactic progesterone in preventing recurrence of PND. | n=94  Pregnant women who had a history of PND severe enough to require medical treatment. | 100mg progesterone intramuscularly immediately after labour and for 7 days following. Then 400mg progesterone suppositories twice daily for 2 months or until the return of menstruation. | Recurrence – no measure described. | Recurrence of PND was 10% (n=9) vs 68% in 221 women who had PND in a previous trial of the same author (Dalton 1982). |
| Marangell et al. 2004; USA | Case series; postnatal; PND | To evaluate the effectiveness of omega-3 fatty  acid supplementation in preventing recurrence of PND. | n=7  Pregnant women who were: (1) at least 18 years old; (2) had a history of PND; and (3) were not currently experiencing a depressive episode.    Exclusion criteria:  (1) depression at time of interview; (2) taking psychotropic medications 2 weeks before the intervention; (3) had a history of not responding to 2 or more antidepressants; (4) had a serious comorbid illness; or (5) posed a danger to themselves or others. | 2,960 mg of fish oil (EPA and DHA) per day between the 34th to 36th week of pregnancy through to study completion at 12 weeks postpartum. | Recurrence of PND - 28-item Hamilton Rating Scale for Depression (HAM-D) and EPDS. | 4 of 7 participants had a depressive episode within 12 weeks, and the study was suspended.    The study failed to demonstrate evidence for the effectiveness of omega-3 fatty acid supplementation in preventing a recurrence of PND. |
| Vaz et al. 2017; Brazil | RCT nested in cohort; antenatal; PND | To evaluate the  efficacy of n-3 PUFAs supplementation (fish oil) on the prevention of PND. | n=60  Cohort inclusion criteria:  (1) 5-13 weeks pregnant; (2) 20-40 years old; (3) no chronic diseases (other than obesity); (4) residing  in the study catchment area; and (5) receiving prenatal care in a public health centre.    For the RCT specifically:  (1) History of depression; or (2) EPDS score ≥9 at baseline.    Exclusion criteria: (1) Experiencing current depression; (2) experiencing psychotic symptoms; (3) reporting a history of mania or hypomania; (4) at risk of suicide; (5) taking psychiatric medications; (6) seeing a psychologist or psychiatrist. | Daily fish oil capsules (1.08 g Eicosapentaenoic and 0.72 g  Docosapentaenoic acids) from week 22-24 of pregnancy and for 16 weeks. | Prevalence of depressive symptoms - EPDS scores ≥11.  Mean and changes of EPDS score from baseline to conclusion of intervention. | There were no differences between intervention and control groups in the prevalence of EPDS ≥11 at any time.  There were no differences in EPDS scores over time, or in changes in EPDS scores from pregnancy to postpartum.    On subgroup analysis, women with a history of depression experienced a significant reduction in EPDS score from pregnancy to postpartum with fish oil compared to placebo. |
| Wisner et al. 1994; USA | Open clinical trial; postnatal; PND | To test the effectiveness of antidepressant medication during the postnatal period to prevent recurrence of PND. | n=23  Asymptomatic pregnant women with at least one past episode of nonbipolar postnatal depression without psychotic features. Women with a history of psychosis or bipolar disorder were excluded. | The treatment group received postpartum monitoring and prophylactic antidepressants - either the antidepressant that had been effective in treating the last episode of PND or Nortriptyline. Participants received the first dose within 24 hours of birth. The control group received monitoring alone. | Recurrence of PND - psychiatric examination and the Inventory to Diagnose Depression. | 6.7% of women who received medication as well as monitoring experienced a recurrence of PND compared to 62% of women who received monitoring alone. |
| Wisner et al. 2001, USA | RCT; postnatal; PND | To examine differences in rate of PND recurrence, time to recurrence, and levels of subclinical symptoms of depression in women taking prophylactic nortriptyline vs placebo. | n=56  Inclusion criteria: (1) Nondepressed since conception of the index pregnancy; (2) < 35 weeks pregnant; (3) aged 21-45 years; (4) at least one past episode of PND occurring within the first 3 months after a live birth; (5) at least one past episode of PND within 5 years prior to study enrolment.    Exclusion criteria: (1) Chronic depression; (2) antidepressant exposure after first trimester; (3) meeting criteria for any other Axis I diagnosis (except generalised anxiety or panic disorder) or antisocial or borderline personality disorder; (4) past episodes of psychosis or bipolar; (5) continuing psychotherapy or taking other psychotropic medications. | Nortriptyline (20-75mg daily) vs placebo. Treatment began immediately after birth. From week 17, the drug was tapered at 33% per week, and discontinued at 20 weeks postpartum. Follow-up continued until one year postpartum. | Recurrent PND - (1) HAM-D; (2) Research Diagnostic Criteria; and (3) psychiatric assessment by principal investigator and a psychiatrist not affiliated with the study.    Time to recurrence - days  Subclinical levels of depression - HAM-D | No difference was found in rate of recurrence, time to recurrence or difference in levels of subclinical symptoms between the nortriptyline and placebo groups. |
| Wisner et al. 2004; USA | RCT; postnatal; PND | To evaluate the impact of prophylactic sertraline on rate of PND recurrence and time to recurrence. | n=22  Subjects were: (1) pregnant <35 weeks; (2) aged 21–45 years; (3) with normal thyroid studies and a complete blood count. They had experienced at least one episode of PND within the last 5 years but were not depressed at the start of the intervention.    Exclusion: (1) Continuation of psychotherapy and/or psychotropic medication after the first trimester; (2) experience of psychosis, bipolar, personality disorder, or any other Axis I diagnosis (except  generalized anxiety or panic disorder). | A 17-week trial of sertraline (a selective serotonin reuptake inhibitor or SSRI) or placebo immediately  after birth.  Dosage was gradually increased to 75mg per day for 12 weeks. From week 17, the dose was tapered over 3 weeks and discontinued at week 20. | PND recurrence – HAM-D and psychiatric assessment. | Rate of recurrence was significantly different between the two groups. 7% of women taking Sertraline (n=1 of 14), and 50% of women in the placebo group (n=4 of 8) experienced recurrence.    Time to recurrence was also significantly longer in the treatment group compared to the placebo group. |

## Table 2: Psychological and/or behavioural interventions

| **Study; country** | **Study design; period; prevention focus** | **Hypothesis/aim** | **Sample (n); participant characteristics** | **Intervention description** | **Outcomes and measures** | **Findings** |
| --- | --- | --- | --- | --- | --- | --- |
| Dimidjian et al., 2015; USA | Case series; antenatal; depression | To examine the feasibility, engagement and clinical outcomes (changes in depression symptom levels and rates of relapse/recurrence) associated with Mindful-Based Cognitive Therapy - Perinatal Depression (MBCT-PD) in pregnant women with a history of major depressive disorder (MDD). | n=49  Inclusion criteria: Pregnant women who: (1) were up to 32 weeks pregnant; (2) met the criteria for past MDD but currently well (EPND <9); (3) were available to attend the group intervention; and (4) were 18 years or older.    Exclusion criteria: (1) MDD in the last 2 months; (2) experiencing another psychiatric disorder; (3) at risk of suicide or homicide; and (4) high-risk pregnancy, e.g., pre-term labour or placental abnormality. | 8 x 2-hour sessions of mindfulness-based cognitive therapy adapted for the perinatal period.  Participants learnt formal and informal mindfulness, and cognitive behavioural skills (e.g., monitoring thoughts, beliefs and emotions; identifying warning signs of relapse; and developing  action plans). A monthly follow-up class was also available. | Satisfaction with intervention - Client Satisfaction Questionnaire  (Attkisson and Zwick 1982) (CSQ-8).  Engagement - class attendance and use of daily practices at home.  Depression symptom severity - EPDS.    Relapse/recurrence - Longitudinal Interval Follow-up Evaluation (LIFE). | Retention was high and participants reported interest, engagement and satisfaction with the program.  Most participants found the program changed how they responded to intense emotions and helped them recognise and respond to early warning signs of relapse/recurrence.    A significant improvement in depressive symptom levels was shown, as well as a relatively low rate of relapse/recurrence (18%). |
| Dimidjian et al. 2016; USA | RCT; antenatal; depression | To evaluate treatment acceptability, participant satisfaction and clinical efficacy (lower rates of relapse/recurrence and lower depressive symptom severity) of MBCT-PD compared to treatment as usual (TAU). | n=86  Inclusion criteria: Pregnant women who: (1) were up to 32 weeks pregnant; (2) met the criteria for past MDD; (3) were available to attend the group intervention; and (4) were 18 years or older.    Exclusion criteria: (1) MDD in the last 2 months; (2) experiencing any other Axis I or II psychiatric disorders that necessitated intervention, e.g., psychosis, eating disorder, or substance abuse; and (3) high-risk pregnancy, e.g., potential for pre-term labour or placental abnormality. | 8 x 2-hour weekly sessions of mindfulness-based cognitive therapy adapted for the perinatal period.    Participants studied formal and informal mindfulness,  cognitive behavioural skills, psychoeducation, yoga and self-care.  A monthly follow-up class was also available. | Acceptability - session attendance and completion of at-home tasks.    Participant satisfaction - 8-item self-report Client Satisfaction Questionnaire (Attkisson & Zwick, 1982).    Relapse/recurrence - Longitudinal Interval Follow-up Evaluation (LIFE; Keller et al., 1987).    Depression symptom severity - EPDS. | Acceptability and participant satisfaction was significantly higher for MBCT-PD than treatment as usual.    Compared to TAU, MBCT-PD participants experienced significantly lower rates of depressive relapse/recurrence (18.4% MBCT-PD vs 50.2% TAU).    MBCT-PD participants also experienced significantly lower depressive symptom severity. |
| Lewis et al. 2021; USA | RCT; postnatal; depression | To evaluate the efficacy of an exercise intervention and a wellness/support intervention compared to usual care in preventing perceived stress and postnatal depression in women with a history of depression. | n=450  Women who: (1) were four weeks postpartum on average; (2) experienced depression before pregnancy; and (3) exercised < 60 mins per week.    Exclusion criteria: (1) no history of depression; (2) unwilling to be randomised to any study condition; (3) < 18 years old; (4) exercising > 60 mins per week at start of intervention; (5) participating in another exercise or weight management study; (6) had another person in the household participating in the study; (7) had a medical condition, illness or took medication that would make exercise unsafe or unwise; (8) unable to exercise for 20 mins continuously; (9) hospitalisation for a psychiatric disorder in the past 6 months; and (10) receiving antidepressants or psychotherapy for depression. | (1) 6-month exercise intervention involving 11 telephone-based sessions; (2) 6-month wellness/support intervention of 11 telephone-based sessions (e.g., promoting sleep, healthy eating, reducing stress); and (3) usual care. | PND - Structured Clinical Interview for DSM-IV Axis I Disorders (SCID-I).    Symptoms of depression - EPDS.    Perceived stress - 14-item Perceived Stress Scale (PSS-14). | Rate of recurrence for participants combined was low (2.4% at 6 months and 3.6% at 9 months). The rate of recurrence was not significantly different between the three study groups.  Median symptoms of depression at 6 months were significantly lower for wellness/support participants compared to usual care participants.  Perceived stress at 6 months was significantly lower for exercise group participants compared to usual care participants and wellness/support participants.  At 9 months, there were no significant differences between the groups in rates of recurrence, symptoms of depression or perceived stress. |
| Molenaar et al. 2020; Netherlands | RCT; antenatal; depression | To evaluate the efficacy of Preventive  Cognitive Therapy (PCT) in preventing perinatal depression (up to 3 months postpartum) while discontinuing antidepressants, compared to continuation of antidepressants over the same period. | n=44  Inclusion criteria: (1) 12-16 weeks pregnant; (2) a history of MDD; (3) currently well; and (4) taking an antidepressant.    Exclusion criteria: (1) multiple pregnancy; (2) insufficient Dutch or English; (3) severe medical conditions requiring urgent care; (4) a history of or current bipolar disorder, mania or hypomania; (5) current self-harm or suicidality; (6) a history of or current psychotic disorder; (7) current drug or alcohol misuse; or (8) current psychological treatment equal to more than once a week for depressive symptoms. | Women allocated to the treatment group received 8 weekly PCT sessions of approximately 30 mins via a telehealth app. They were also supported by their psychiatrist or general practitioner to taper and cease their antidepressant over a four-week period.    PCT uses techniques focused on dysfunctional beliefs and schema using cognitive challenging techniques.    The control group was instructed to continue seeing their prescribing clinician as usual.    Women were followed during pregnancy and up to 3 months postpartum. | Relapse or recurrence of depression - Structured Clinical Interview for DSM-IV Axis I Disorders (SCID-I) and the HAM-D.    Symptoms of depression - EPDS.    Symptoms of anxiety - State-Trait Anxiety Inventory (STAI). | There was no significant difference in the risk of relapse between the treatment group (receiving PCT with gradual cessation of antidepressant) and the control group (remaining on antidepressant therapy). |

## Table 3: JBI Checklist for Case Series (Munn, Barker et al. 2020)

|  | **Dalton (1985)** | **Marangell, Martinez et al. (2004)** | **Dimidjian, Goodman et al. (2015)** |
| --- | --- | --- | --- |
| - Were there clear criteria for inclusion in the case series? | Yes | Yes | Yes |
| - Was the condition measured in a standard, reliable way for all participants included in the case series? | Unclear | Unclear | Yes |
| - Were valid methods used for identification of the condition for all participants included in the case series? | No | Yes | Yes |
| - Did the case series have consecutive inclusion of participants? | Unclear | Unclear | Unclear |
| - Did the case series have complete inclusion of participants? | Unclear | Unclear | Unclear |
| - Was there clear reporting of the demographics of the participants in the study? | No | Yes | Yes |
| - Was there clear reporting of clinical information of the participants? | No | Yes | Yes |
| - Were the outcomes or follow up results of cases clearly reported? | Yes | Yes | Yes |
| - Was there clear reporting of the presenting site(s)/clinic(s) demographic information? | No | No | Yes |
| - Was statistical analysis appropriate? | No | N/A | Yes |

## Table 4: JBI Critical Appraisal Tool for Assessment for Randomised Controlled Trials (Barker, Stone et al. 2023)

|  | **Vaz, Farias et al. (2017)** | **Wisner, Perel et al. (2001)** | **Wisner, Perel et al. (2004)** | **Dimidjian, Goodman et al. (2016)** | **Lewis, Schuver et al. (2021)** | **Molenaar, Brouwer et al. (2020)** |
| --- | --- | --- | --- | --- | --- | --- |
| **Internal validity** | | | | | | |
| Bias related to selection and allocation | | | | | | |
| Was true randomization used for assignment of participants to treatment groups? | Yes | Yes | Yes | Yes | Yes | Yes |
| Was allocation to treatment groups concealed? | Yes | Yes | Yes | N/A | No | N/A |
| Were treatment groups similar at the baseline? | Yes | Yes | Yes | Yes | No | Yes |
| Bias related to administration of intervention/exposure | | | | | | |
| Were participants blind to treatment assignment? | Yes | Yes | Yes | N/A | Yes | N/A |
| Were those delivering the treatment blind to treatment assignment? | Yes | Yes | No | N/A | N/A | N/A |
| Were treatment groups treated identically other than the intervention of interest? | Yes | Yes | Yes | Yes | Yes | Yes |
| Bias related to assessment, detection and measurement of the outcome | | | | | | |
| Were outcome assessors blind to treatment assignment? | Unclear | No | Yes | Yes | Unclear | Yes |
| Were outcomes measured in the same way for treatment groups? | Yes | Yes | Yes | Yes | Yes | Yes |
| Were outcomes measured in a reliable way? | Yes | Yes | Yes | Yes | Yes | Yes |
| Bias related to participant retention | | | | | | |
| Was follow up complete and if not, were differences between groups in terms of their follow up adequately described and analysed? | Yes | Yes | Yes | Yes | No | Yes |
| **Statistical Conclusion Validity** | | | | | | |
| Were participants analysed in the groups to which they were randomized? | Yes | Yes | Yes | Yes | Yes | Yes |
| Was appropriate statistical analysis used? | Yes | Yes | Yes | Yes | Yes | Yes |
| Was the trial design appropriate and any deviations from the standard RCT design (individual randomization, parallel groups) accounted for in the conduct and analysis of the trial? | Yes | Yes | Yes | Yes | Yes | Yes |

## Table 5: JBI Critical Appraisal Checklist for Quasi-Experimental Studies (Tufanaru, Munn et al. 2017)

|  | **Wisner and Wheeler (1994)** |
| --- | --- |
| 1. Is it clear in the study what is the ‘cause’ and what is the ‘effect’ (i.e. there is no confusion about which variable comes first)? | Yes |
| 1. Were the participants included in any comparisons similar? | Unclear |
| 1. Were the participants included in any comparisons receiving similar treatment/care, other than the exposure or intervention of interest? | Yes |
| 1. Was there a control group? | Yes |
| 1. Were there multiple measurements of the outcome both pre and post the intervention/exposure? | Yes |
| 1. Was follow up complete and if not, were differences between groups in terms of their follow up adequately described and analysed? | Yes |
| 1. Were the outcomes of participants included in any comparisons measured in the same way? | Yes |
| 1. Were outcomes measured in a reliable way? | Yes |
| 1. Was appropriate statistical analysis used? | Yes |

## Table 6: Cochrane risk-of-bias tool for randomized trials (RoB 2) (Sterne, Savović et al. 2019)

|  | **Vaz, Farias et al. (2017)** | **Wisner, Perel et al. (2001)** | **Wisner, Perel et al. (2004)** | **Dimidjian, Goodman et al. (2016)** | **Lewis, Schuver et al. (2021)** | **Molenaar, Brouwer et al. (2020)** |
| --- | --- | --- | --- | --- | --- | --- |
| ROB arising from the randomisation process | Low | Low | Low | Low | Low | Low |
| ROB due to deviations from the intended interventions (effect of assignment to intervention) | Low | Low | Low | Low | Low | Low |
| ROB due to deviations from the intended interventions (effect of adhering to intervention) | Low | Low | Low | Low | Low | Low |
| Missing outcome data | Low | Low | Low | Low | Some concerns | Low |
| *What is the predicted direction of bias due to due to missing outcome data?* | N/A | N/A | N/A | N/A | Unpredictable | N/A |
| ROB in measurement of the outcome | Low | Low | Low | Low | Low | Low |
| ROB in selection of the reported result | Low | Low | Low | Low | Low | Low |
| **Overall risk of bias** | **Low** | **Low** | **Low** | **Low** | **Low** | **Low** |

## Table 7: The Risk Of Bias In Non-randomized Studies – of Interventions (ROBINS-I) assessment tool (Sterne, Hernán et al. 2016)

|  | **Dalton (1985)** | **Marangell, Martinez et al. (2004)** | **Wisner and Wheeler (1994)** | **Dimidjian, Goodman et al. (2015)** |
| --- | --- | --- | --- | --- |
| Bias due to confounding | Moderate | Moderate | Moderate | Moderate |
| *What is the predicted direction of bias due to confounding?* | Unpredictable | Unpredictable | Unpredictable | Unpredictable |
| Bias in selection of participants into the study | Low | Low | Low | Low |
| Bias in classification of interventions | Low | Low | Low | Low |
| Bias due to deviations from intended interventions | Low | Low | Low | Moderate |
| *What is the predicted direction of bias due to deviations from the intended interventions?* | N/A | N/A | N/A | Unpredictable |
| Bias due to missing data | Low | Low | Low | Low |
| Bias in measurement of outcomes | Moderate | Low | Low | Low |
| *What is the predicted direction of bias due to measurement of outcomes?* | Favours experimental | N/A | N/A | N/A |
| Bias in selection of the reported result | Low | Low | Low | Low |
| **Overall risk of bias** | **Moderate** | **Moderate** | **Moderate** | **Moderate** |
| *What is the overall predicted direction of bias for this outcome?* | *Favours experimental* | *Unpredictable* | *Unpredictable* | *Unpredictable* |
